# A Novel LTE Scheduling Algorithm for Green Technology in Smart Grid

**DOI:** 10.1371/journal.pone.0121901

**Published:** 2015-04-01

**Authors:** Mohammad Nour Hindia, Ahmed Wasif Reza, Kamarul Ariffin Noordin, Muhammad Hasibur Rashid Chayon

**Affiliations:** Department of Electrical Engineering, Faculty of Engineering, University of Malaya, Kuala Lumpur, Malaysia; Tianjin University of Technology, CHINA

## Abstract

Smart grid (SG) application is being used nowadays to meet the demand of increasing power consumption. SG application is considered as a perfect solution for combining renewable energy resources and electrical grid by means of creating a bidirectional communication channel between the two systems. In this paper, three SG applications applicable to renewable energy system, namely, distribution automation (DA), distributed energy system-storage (DER) and electrical vehicle (EV), are investigated in order to study their suitability in Long Term Evolution (LTE) network. To compensate the weakness in the existing scheduling algorithms, a novel bandwidth estimation and allocation technique and a new scheduling algorithm are proposed. The technique allocates available network resources based on application’s priority, whereas the algorithm makes scheduling decision based on dynamic weighting factors of multi-criteria to satisfy the demands (delay, past average throughput and instantaneous transmission rate) of quality of service. Finally, the simulation results demonstrate that the proposed mechanism achieves higher throughput, lower delay and lower packet loss rate for DA and DER as well as provide a degree of service for EV. In terms of fairness, the proposed algorithm shows 3%, 7 % and 9% better performance compared to exponential rule (EXP-Rule), modified-largest weighted delay first (M-LWDF) and exponential/PF (EXP/PF), respectively.

## Introduction

Nowadays, the existing power grid systems are facing difficulties to cope up with increasing demand for energy, leading to a lack of reliability, instability and poor *quality of service* (QoS) [1]. Therefore, recent researches focused on upgrading the traditional grid towards smart grid (SG) system [2–4]. The enhancement and modernization of the present grid to an SG structure can be accomplished by providing two-way communication between the control unit and the distributed components all over the network. SG can guarantee a better efficiency in terms of customer services, power management, flexibility and failure prediction, and it can offer less expensive services than the traditional system. Moreover, SG offers a large amount of support for the environment by integrating the renewable energy into the grid [5, 6]. By using certain management algorithms, it can help the existing system to store the surplus electricity during the off peak period in the Vehicle-to-Grid (V2G) nodes, then reuse it when necessary. Hundreds of thousands of nodes, such as V2G can be used as storage devices for power generation from solar or wind by using V2G batteries. This integration offers consistent, controllable, stable and reliable power supply. In addition, it helps minimizing the damages caused by the present grid system on the environment by decreasing the CO_2_ emission and fuel consumption [7–10]. However, renewable energy has its own drawbacks, such as huge dependency of solar cell and wind energy generator on the weather condition, thus the instable power generation disrupts the grid system. As proposed in [11], V2G or electrical vehicle node can add on average 560–910 Watt to the grid based on the battery size. Large number of vehicles contributes to a significant amount of energy which helps in stabilizing the power grid. The V2G system provides dual benefits for both the electricity provider and the consumer. Electrical vehicle (EV) owners can sell their excessive stored energy to the grid as a distributed generator so that the grid can purchase when it needs. Thus, the dependency on regular generators, such as nuclear and fuel power generators decreases significantly.

To provide smooth running of EV over the grid, an aggregator and two SG applications are required, which are distribution automation (DA) and distributed energy system-storage (DER). The aggregator is defined as a bridge between the vehicles and the control unit that contains the information about the number of EVs and their coordination on the map. It also provides the status of charging and discharging, amount of power consumption and generation to the grid and billing information service to the users. Whereas, the DA application acts as a connection between the end user and the transmission system. Moreover, it remotely controls, monitors and repairs the electrical distributed components on the grid. In addition, it has a major role in terms of minimizing the power shortage, provides high reliability of electrical power consumption, and high ability to control and balance the load on the grid. DA application can be considered as a real time application since the feedbacks have strict requirements in terms of delay [12]. Finally, DER supplies the required energy during the peak loads and stores the surplus power when the demand is low. This can be a consistent, controllable, and reliable energy source [13].

In order to allow the integration between the green technology and SG system, so that the whole system can benefit from the above mentioned advantages, three different groups are required to work in harmony as one group to complete this mission successfully. These groups and their roles can be described as follows: firstly, the power engineers play major role in the energy conversion process. Secondly, the electrical engineering group proposes a technique to guarantee the exact voltage and current flowing from the V2G to grid, and via versa. Finally, the communication engineering group adds the communication layer that secures the energy flow and data exchange between the vehicle and the public grid as well as guarantees the QoS’ demands of end-to-end users and applications.

As using of game theory (cooperative and non-cooperative techniques) increases dramatically nowadays, integration with SG applications can enhance the system performance, such as fairness index as well as the throughput since it has the ability of dynamic resource allocation among application users [14]. Some researchers [15] introduced a weighting structure into the spatial prisoner’s dilemma game to analyze the cooperative behaviors. Three types of weight distributions were considered, namely exponential, power-law and uniform distributions. This mechanism showed an enhancement in terms of the cooperators frequency because of the high heterogeneity of link strength.

Moreover, in [16], the authors considered the size of the interaction neighborhood to find out the evolution of cooperation on a square lattice in the prisoner’s dilemma game. Their work was based on investigating the effects of noise and the cost-to-benefit ratio. The results showed that the cooperation reached a climax as the noise increased. The authors demonstrated that the cooperation was remarkably enhanced by increasing the size of interaction neighborhood. It has been reported that the cooperation is faded when the size of interaction neighborhood becomes too large.

Besides, a spatial prisoner’s dilemma game model is studied in [17] to analyze the impact of separation between the interaction and learning neighborhood. The authors considered two different cases where the size of one neighborhood was fixed and the other one was varied and vice versa. The results showed that this separation strongly influenced the cooperative behaviors among players. According to their findings, medium-sized neighborhood can manage and assist the cooperation among individuals on the square lattice when compared to the standard case.

From the communication engineering aspect, distributed components, such as generators, power transfers and distribution feeders should be supported by a bidirectional communication network that is fast, reliable and secure communication technology. Several wireless technologies can be employed in SG applications, such as 3G cellular network, Worldwide Interoperability for Microwave Access (WiMAX) and Long Term Evolution (LTE). However, according to laboratory and field tests [18], LTE network meets all the technical requirements for SG communications because it offers reliability, very low latency, high data rate and spectral efficiency, as well as commercial advantages, but it is not specifically designed for SG applications. As the scheduling mechanism has an essential impact on the performance of LTE network, a new scheduling algorithm must be proposed or the existing algorithms need to be optimized to satisfy the demands of SG application.

Uplink and downlink scheduling are separated in LTE and the scheduling decisions can be taken independently of each other. Orthogonal frequency division multiple access (OFDMA) technique is deployed in LTE for downlink stream. Its robustness and reliability against multi fading, interference and higher spectral efficiency are proved [19, 20]. Whereas, single carrier FDMA (SC-FDMA) is utilized for uplink stream because of its power conservation at the user equipment (UE). The scheduler takes into account the channel quality inductor (CQI), which is updated regularly at each transmission time interval (TTI). Resource Blocks (RBs) are assigned by the scheduler to users at each 1 ms of scheduling interval [21]. For further scheduling improvements of real time applications, several algorithms, such as exponential/PF (EXP/PF), modified-largest weighted delay first (M-LWDF) and exponential rule (EXP-Rule) schemes have been proposed. These schemes improve the scheduling performance in terms of throughput, latency and fairness. It is worth to mention that, these approaches are recognized as channel and QoS aware strategies.

In this paper, three SG applications for renewable energy are investigated in order to study their applicability in LTE network. LTE network is not specially designed to accommodate these applications. Therefore, we propose a novel bandwidth estimation and allocation technique and a new scheduling algorithm to make the LTE network fully compatible with these applications. The bandwidth estimation and allocation technique is used for resource allocation based on the priority of each application. By using this method, it is possible to guarantee that each application will be served by the optimized amount of RBs. As a result, the network resources will be used efficiently. Furthermore, suggested scheduling algorithm provides a dynamic scheduling solution for different types of applications. This algorithm is able to serve the users’ applications according to the preference of each application. Moreover, it prioritizes users for service based on three criteria, namely, packet waiting delay, past average throughput and instantaneous transmission rate. Based on the authors’ knowledge, this is the first time, these applications have been studied from the telecommunication side and being optimized for integration with an LTE network.

The rest of the paper is organized as follows. Section 2 describes the related work on communication methods of V2G and different popular scheduling algorithms. Section 3 discusses about the system model and Section 4 illustrates the results and discussion. After stating the limitations and future work in Section 5, conclusion is presented in Section 6.

## Related Work

### V2G

The issues related to implementing the V2G, i.e., electronic vehicles (EV) with ability to connect with and imply the request from the central control, smart aggregator with bidirectional communication system, intelligent metering with fast and reliable communication technique between the central control module and the end user need to be solved. From the telecommunication side, these difficulties are summarized as enhancing the efficiency of data exchange between the nodes and the applications in terms of throughput, packet loss ratio and delay. Wencong Su and Wente Zeng [22] describe three possible communication protocols for V2G, such as HomePlug, ZigBee and cellular network. HomePlug uses broadband communications over low-voltage power line which can transfer data at a maximum rate of 14 Mbps, higher than other two technologies. This protocol does not need additional battery and free from all issues regarding wireless data transmission. On the other hand, ZigBee is for small and self-programming mesh network devices (i.e., smart meter, electric vehicle charging station or personal electric vehicle) based on IEEE 802.15.4 wireless standard. Its maximum data rate is 250 kbps at 2.4 GHz, which satisfies the EV application’s requirements. The authors [22] mention that, Bluetooth and Z-wave technologies can be used in future for further improved result. According to the authors [22], cellular network can be used for highly mobile devices in case of long-range wireless communication. Cellular network requires more power consumption to enable long-range transmission and the data rate is above 100 kbps, which satisfies the requirements of EV applications. Commercial cellular services offer sufficient capabilities to communicate billing information and it may be a feasible option at public charging facilities.

Active Network Management (ANM) is used in [23, 24] based on IEEE supervisory control and data acquisition standard (IEEE SCADA), which is designed to run over a serial line-either in point-to-point or in the multi-drop system. In [25], the authors propose wireless sensor network by using IEEE 802.15.4 (CC2420 radio Chips) in an SG environment to communicate between the smart meter and the control room. Sensors remotely read the meter and send the data to the control panel. They also include substations, power control room, and underground network transformer vault.

Wireless mesh [13] is another viable option to use in advanced metering infrastructure (AMI) and home energy management. This technology is cost effective with dynamic self-organization, self-configuration, self-healing, and high scalability services, which provides improved network performance, load balancing of the network and extended network coverage. However, mesh network needs a third party company to manage the network and it is less secured and it causes additional overheads in the communication channel that would result in a reduced available bandwidth.

Power line communication (PLC) [26] can be well suited to home area network (HAN) applications and urban areas for SG applications, such as smart metering, monitoring and control applications because of its cost effectiveness, ubiquitous nature and wide availability of PLC infrastructures. The low-bandwidth characteristics (20 kbps for neighborhood area network), inability to handle different types and a large number of devices, poor performance in long distance communication and hugely affected by PLC environment are the major drawbacks of the packet loss rate system, which can be overcome by combining with other technologies, i.e., general packet radio service and global positioning system.

Digital subscriber lines (DSLs) use wires of the existing voice telephone network [13, 26]. Main features of DLSs are the high bandwidth data transmission, low cost and widespread availability. Although due to the high installation cost of fixed infrastructure, it is difficult to implement is rural areas. Moreover, lack of standardization and distance dependence may cause more problems. Table 1 illustrates the characteristics, limitations, and possible applications of different communication technologies [27, 28].

**Table 1:** Comparison of different communication technologies.

Technology	Speed	Coverage Area	Frequency Operation	Limitations	Applications
Bluetooth (802.15.1)	3 Mbps	1–100 m	2.4–2.48 GHz	Short range, high interference	AMI, HAN
WiFi (802.11n)	300 Mbps	100 m	2.4–5.4 GHz	Short range, high interference	AMI, HAN
ZigBee	250 Kbps	75 m	2.4 GHz	Low data rate, Short range, high interference	AMI, HAN
Z-Wave	40 Kbps	30 m	EEUU: 908.42 MHz Europa: 868.42 MHz	Low data rate, Short range	AMI, HAN
GPRS	Up to 170 Kbps	1–10 km	900–1800 MHz	Low data rate	AMI, Demand Response, HAN
3G	384 Kbps- 2 Mbps	1–10 km	1.92–1.98 GHz 2.11–2.17 GHz (Licensed)	Costly spectrum fees	AMI, Demand Response, HAN
WiMAX	Up to 75 Mbps	10–50 km (LOS) 1–5 km (NLOS)	2.5 GHz, 3.5 GHz, 5.8 GHz	Not widespread	AMI, Demand Response
PLC	2–3 Mbps	1–3 km	1–30 MHz	Harsh and noisy channel environment	AMI, Fraud Detection

*Table adapted from the source files of*: [27] and [28].

### LTE scheduling algorithm

Each scheduling algorithm has different methods to determine the users’ scheduling priority, such as buffer status, delay, expected throughput, channel status and past average throughput. The principal aims of the schedulers should be maximizing the throughput, providing good QoS to the user and providing good fairness to the non-real time (N-RT) user. This paper focuses on the most popular algorithms, namely proportional fairness (PF), M-LWDF, EXP/PF and EXP-Rule to analyze the performance with the proposed algorithm.

In [29], PF is proposed to provide service for N-RT. The scheduling metric is based on prioritizing the user with maximum relative channel quality indicator (RCQI) which is defined as the ratio between the instantaneous data rate supported by the user on the CQI value and the average data rate of previous transmission till the present TTI. However, PF does not consider the delay metric, that is why it cannot serve the real time (RT) application.

Many algorithms have been proposed as an extension to the PF algorithm, such as EXP/PF [30]. It distinguishes the user depends on their packet type. For RT users, it is emerging, based on the benefits of the exponential function which guarantees the delay boundaries of RT application and at the same time, maximizes the system throughput. The robustness of this algorithm are based on taking the exponential of the end-to-end delay of user’s packets, thus scheduling metric exponentially grows along with delay metric. Whereas, the N-RT users are served as PF with a specific degree of service. This degree is controlled by the proportion of waiting packets of RT applications at BS. The main drawback of this algorithm is the positive probability of drop of the services from N-RT applications as shifting it to RT one.

In [31], the M-LWDF is used in so many fields, for instance, streaming video application; it mixes between the user channel status quality and the time delay of the packets. It proves to be a suitable solution for scheduling flows of the high speed downlink packet access. It is based on the head of line packet delay along with the PF metric to ensure the QoS provision fairness and spectral efficiency among the users. It takes into consideration the channel condition, head of line delay and the packet loss ratio.

EXP-Rule algorithm [32] is considered as a further modification of the EXP/PF algorithm. The main purpose of this algorithm is to distribute radio resources among users in a fair and efficient manner so that the system throughput can be maximized. The EXP-Rule algorithm gives higher priority to the user with more transmission delay besides the channel condition.

## Materials and Methods

**Fig 1:**
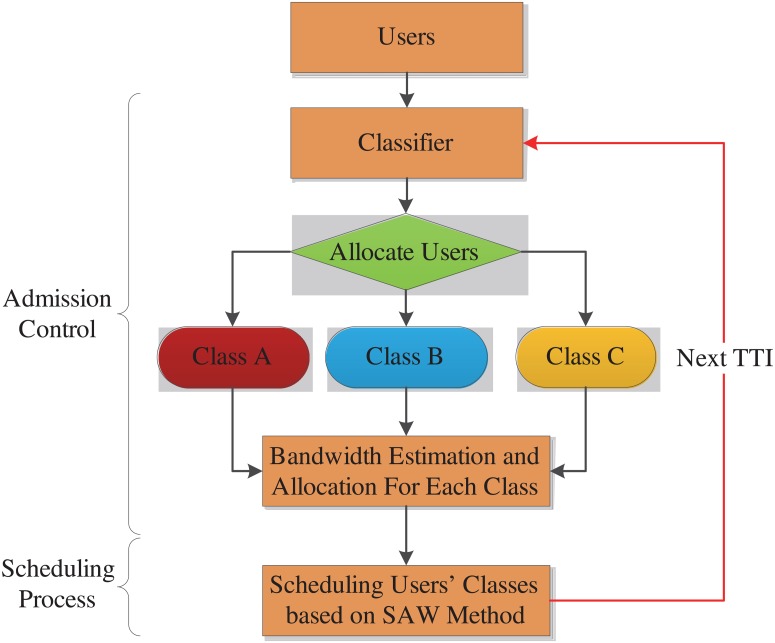
Flow chart of the system model.

As it is illustrated in Fig. 1, the system model is divided into two main levels, namely admission control and scheduling process.

### 2 Admission control

Due to random variation of radio condition in LTE network, the obtainable bit rate for the active user is supposed to vary based on the signal to interference noise ratio (SINR) which is received at the mobile station (MS) from the base station (BS). Allowing or denying the service for the new user of specific class is based on the admission control procedure. Admission control has two steps, which are classifier and bandwidth estimation and allocation. The first step authenticates whether or not the new user belongs to the classes (applications) (Table 2) and the second step decides the acceptance or rejection of new user based on the availability of bandwidth of each class.

**Table 2:** Demands of smart grid applications.

SG Application	Bandwidth Requirement [kpbs]	Delay Budget [ms]
Distribution automation (DA)	100	100
Distributed energy system-storage (DER)	500	300
Electrical vehicle (EV)	64	2000

Each user will be allocated to related class based on its demands (Table 2) by the classifier as follows:

Class 1: Urgent to be served which has so strict delay tolerance, such as DA application (priority 1).

Class 2: Requires an RT service which has a tolerance in terms of delay, such as DER application (priority 2).

Class 3: Requires N-RT service which has a high tolerance in terms of delay, such as EV application (priority 3).

The classifier determines the user number of each class. Then this information will pass to the bandwidth estimation and allocation level to allocate efficient RBs based on its required data rate.

#### Bandwidth estimation and allocation

Several issues control the data transmission ability of RB, such as distance from the BS, power allocation for each RB and the external and internal noise. In our model, it is assumed that, all users have fixed coordination at the map and the same transmission power is allocated for all sub-carriers. Moreover, two types of noises are defined which are internal and external noise. Thermal noise at the receiver end is internal noise, whereas the interference from neighboring BSs is defined as external noise. The SINR is calculated by multiplying the channel gain of user *i* RB *j* (*C*_*gain i*,*j*_) (1) with assigned power for the subcarriers (*P*_*subcarrier*_) over the noise (2) [33].

$${}_{Cgain\;i,j=10}\frac{pathloss}{10}+\frac{multi-fading}{10}+\frac{fading}{10}$$ (1)

$$SINR=\frac{C_{gaini,j}\times P_{subcarrier}}{N_{0}+I}$$ (2)

where *pathloss*, *multi-fading* and *fading* are measured in scale of dB, *N*_*0*_ is the thermal noise and *I* is the interference from surrounding BSs.

At each TTI, MS reports their instantaneous downlink SINR to BS that is used to determine the ability of data transmission for allocatable RB *j* of user *i* at time *t* ($R_{i}^{j}\left(t\right)$) as follows:

$$R_{i}^{j}\left(t\right)=\frac{n-bits}{symbol}\times \frac{n-symbols}{slot}\times \frac{n-slots}{subcarrier}\times \frac{n-subcarrier}{RB}$$ (3)

where *n—bits*, *n—symbols*,*n—slots* and *n—subcarrier* are the number of bits, number of symbols, number of slots and number of subcarriers, respectively [34].

From (3), it can be said that the number of bits per symbol (*n*-*bits*) has a strong impact on RB’s obtainable data rate. Based on the bit per symbol, the data rate will be changed at each TTI along with SINR value. Total number of RBs ($n_{i}^{c}$) required to satisfy the user *i* of class *c* can be obtained by the ratio of the demand data rate (*r* ^*c*^) and obtainable data rate for RB ($R_{i}^{j}\left(t\right)$) with specific modulation and coding scheme (MCS) (4). Then the number of required RBs at time *t* for user *i* is calculated by (5). All sets of RBs, which are assigned to user *i* at time *t* must have the same MCS and one RB cannot be assigned to more than one user at time *t*.

$$n_{i}^{c}=\frac{r^{c}}{R_{i}^{j}\left(t\right)}$$ (4)

$$NRB_{i}^{c}\left(t\right)=\frac{n_{i}^{c}}{\tau_{i}}$$ (5)

where $NRB_{i}^{c}\left(t\right)$ is the number of RBs required for user *i* of class *c* at time *t* and *τ*_*i*_ is the maximum delay budget.

For providing services to the user, scheduler requires to know the exact number of RBs required per user for each class which is calculated by (5). After that, in terms of measuring the required bandwidth for class *c* at time *t*, (6) is proposed.

$$R^{c}\left(t\right)=N^{c}\left(t\right)\times b^{c}$$ (6)

where *R* ^*c*^ (*t*)is the required bandwidth for class *c* at time *t*,*N* ^*c*^ (*t* ^*c*^) is the number of users allocated to class *c* at time *t* and *b* ^*c*^is the required data rate.

The bandwidth estimation is followed by the bandwidth allocation procedure. The main purpose of bandwidth allocation is to determine whether or not there are enough resources to cover the demand of a particular class. Otherwise, the resources will be shifted from the lower priority class to the higher priority class to cover the shortage and it will be repeated until all network resources have been utilized. For instance, if a new user comes to class 1 and the resources of class 1 are not enough to satisfy that user, then resources from class 3 will be shifted to class 1. High priority class users will be served until all the resources are assigned. This algorithm (Table 3) will be updated and calculated at each TTI.

**Table 3:** Proposed algorithm of bandwidth estimation and allocation.

***Algorithm 1***. *Bandwidth allocation*
1:**procedure** bandwidth allocation for each class
2:insert *A*, *B* ^*c*^(*t*), *mc*
3:**for all** *c* such that 1 *≤ c ≤ A* and *c ∈ A* **do**
4:**c**ollect *B* ^*c*^(*t*)
5:compute *R* ^*c*^(*t*) from (11)
6:**if** *B* ^*c*^ (*t*)≥*R* ^*c*^(*t*)
7:serve user *i*, *i =* [1, 2, ……, mc]
8:**else**
9:compute *δ = |B* ^*c*^ (*t*)- *R* ^*c*^ (*t*)|
10:**if** *B* ^*A*^(*t*)≥*δ*
11: *B* ^*A*^ (*t*) = *B* ^*A*^ (*t*) - *δ*
12: *B* ^*c*^ (*t*) = *B* ^*c*^ (*t*) - *δ*
13:**else if** *B* ^*A*^ (*t*) + *B* ^*A-1*^ (*t*) ≥ *δ*
14: *B* ^*A-1*^ = *B* ^*A-1*^-|*B* ^*A*^-*δ*|
15: *B* ^*c*^ (*t*) = *B* ^*c*^ (*t*)+*δ*
16:**else** serve users up to *B* ^*c*^ (*t*)
17:**end for**
18:**end procedure**

where *B* ^*c*^(*t*) is the offered data rate from the system to class *c* at time *t*, *δ* is the difference between the offered and demand data rate, *A* is the total number of classes and *mc* is the set of user belongs to class *c*.

### 3 Proposed scheduling scheme

**Fig 2:**
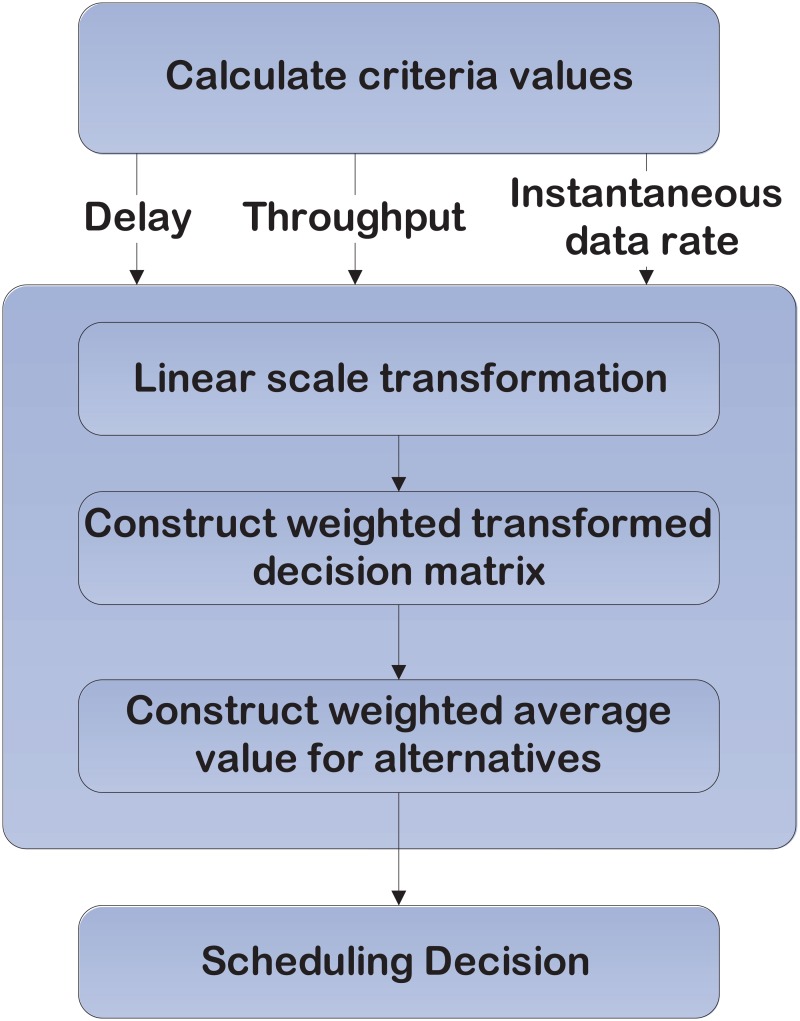
Flow chart of the proposed scheduling algorithm.

The proposed scheduling algorithm is based on simple additive weighting (SAW) technique, which is a multi-attribute decision making method to find out the best option from all feasible alternatives. Our proposed scheduling scheme relies on proportional linear transformation of the raw data (criteria values), thus the relative order of magnitude of the standardized scores remains equal. Moreover, it ensures low complexity, dynamic adjusting and good controlling to the scheduling algorithm behavior as well as it satisfies the demand of SG application. The dynamic weighting factors, namely delay, past average throughput and instantaneous transmission rate are used to adjust the scheduling decision for each application (class). For instance, some applications require high emphasis on the delay, such as DA, thus the higher weight will be given to delay than others. Whereas, the EV requires more emphasis on throughput than delay, thus the higher weight will be given to past average throughput rather than delay or channel status. The proposed scheduling algorithm is described in Fig. 2.

#### Calculation of the criteria values

Three criteria values for each user are calculated and inserted as input to the proposed scheduling algorithm as follows:

a) Delay metric of user *i* is calculated as the ratio of the difference between current time and stamped time of packet at the buffer queue to the delay budget of its related class. And, if the delay factor is bigger than one, user *i* ’s packets will be dropped from further evaluation (7).
  $$D_{i}^{c}\left(t\right)=\frac{t-T_{stamp,i}}{\tau^{c}}$$ (7)
  where $D_{i}^{c}\left(t\right)$ is the delay factor of user *i* for class *c* at time *t*, *T*_*stamp*,*i*_ is the entrance time of user *i’*s packets in the buffer queue and *τ* ^*c*^is the delay budget of class *c*.
b) The instantaneous data rate is the expected data rate, which could be achieved by users *i* from class *c* at time slot *t*.
c) The past average throughput metric is used as pointer to determine the data rate of user *i* in previous TTI. It calculates as a moving average throughput as follows:
  $$\begin{array}{l} {\bar{TH}}_{i}^{c}\left(t\right)=\psi \times {\bar{TH}}_{i}^{c}\left(t-1\right)+\left(1-\psi \right)\times r_{i}^{c}\left(t\right)\\ where\;\text{0}\leq \psi \leq 1 \end{array}$$ (8)
  where ${\bar{TH}}_{i}^{c}\left(t\right)$ is the past average throughput of user *i* of class *c* at time *t*, *Ψ* is constant related to the window size and $r_{i}^{c}\left(t\right)$ is the acquired data rate of user *i* from class *c* at time *t*.

**Step 1**. *Linear scale transformation*. Data collection is normalized by the maximum value of the criterion for all users as a process to unify all values up to one measurement scale for the specific class. Then, it inserts into the normalized decision matrix at time *t* (*R* (*t*)) as follows:

$$\begin{array}{l} D_{i1}^{c}\left(t\right)=\frac{D_{i}^{c}\left(t\right)}{D_{mc}^{*}\left(t\right)},for\;i=1,2,\ldots ,mc\\ r_{i2}^{c}\left(t\right)=\frac{r_{i}^{c}\left(t\right)}{r_{mc}^{*}\left(t\right)},for\;i=1,2,\ldots ,mc\\ {\bar{TH}}_{i3}^{c}\left(t\right)=\frac{{\bar{TH}}_{i}^{c}\left(t\right)}{{\bar{TH}}_{mc}^{*}\left(t\right)},for\;i=1,2,\ldots ,mc \end{array}$$ (9)

$$\begin{array}{l} R\left(t\right)=\left[\begin{array}{ccc} D_{11}\left(t\right) & r_{12}\left(t\right) & {\bar{TH}}_{13}^{c}\left(t\right)\\ \begin{array}{l} D_{21}\left(t\right)\\ \vdots \end{array} & \begin{array}{l} r_{22}\left(t\right)\\ \vdots \end{array} & \begin{array}{l} {\bar{TH}}_{23}^{c}\left(t\right)\\ \vdots \end{array}\\ D_{mc1}\left(t\right) & r_{mc2}\left(t\right) & {\bar{TH}}_{mc3}^{c}\left(t\right) \end{array}\right] \end{array}$$ (10)

where $D_{mc}^{*}\left(t\right)$, $r_{mc}^{*}\left(t\right)$ and ${\bar{TH}}_{mc}^{*}\left(t\right)$ are the maximum value of delay, instantaneous data rate and past average throughput, respectively.

**Step 2**. *Construction of the weighted transformed decision matrix*. In this step, a set of weight coefficients $w_{D}^{c}$,$w_{r}^{c}$ and $w_{\bar{TH}}^{c}$ are accommodated to the transformed decision matrix to build the weighted transformed decision matrix *v* (*t*) as follows:

$$v\left(t\right)=\left[\begin{array}{ccc} w_{D}^{c}\times D_{11}\left(t\right) & w_{r}^{c}\times r_{12}\left(t\right) & w_{\bar{TH}}^{c}\times {\bar{TH}}_{{}_{13}}\left(t\right)\\ \begin{array}{l} w_{D}^{c}\times D_{21}\left(t\right)\\ \vdots \end{array} & \begin{array}{l} w_{r}^{c}\times r_{22}\left(t\right)\\ \vdots \end{array} & \begin{array}{l} w_{\bar{TH}}^{c}\times {\bar{TH}}_{{}_{23}}\left(t\right)\\ \vdots \end{array}\\ w_{D}^{c}\times D_{mc1}\left(t\right) & w_{r}^{c}\times r_{mc2}\left(t\right) & w_{\bar{TH}}^{c}\times {\bar{TH}}_{{}_{mc3}}\left(t\right) \end{array}\right]$$ (11)

**Step 3**. *Construction of the weighted average value for users*. In this process, after summing up the criteria values ($A_{i}^{*}\left(t\right)$) belong to user *i* in (12), the scheduler will serve the user according to the obtained values in descending order.

$$A_{i}^{*}\left(t\right)=\sum_{g=1}^{A}v_{ig}\left(t\right)$$ (12)

where $A_{i}^{*}\left(t\right)$ is the descending order of the users at time *t* and *v*_*ig*_(*t*) is the metric *v* index.

## Results and Discussion

The LTE-Sim is used as a simulation tool, which is based on C++. The testing scenario is a single cell with interference and the simulation inputs are illustrated in Table 4. All the users have fixed location in the cell and their coordination is well known by the scheduler. As a fairness index, the Jain fairness index method is adopted [35]. Two types of loss models are utilized in this scenario, which are path loss (it has a direct relation to the distance from the BS) and shadow fading.

**Table 4:** Simulation parameters.

Parameters	Values
Cell Radius	1 km
Bandwidth	5 MHz
Number of Cell	1
Number of RBs	25
Bearer Types	From QCI 1- QCI 15
Environment Type	Urban
eNodeB Height	20 m
UE Height	1.5 m
eNodeB Tx power	46 dBm
UE Tx power	23 dBm

**Fig 3:**
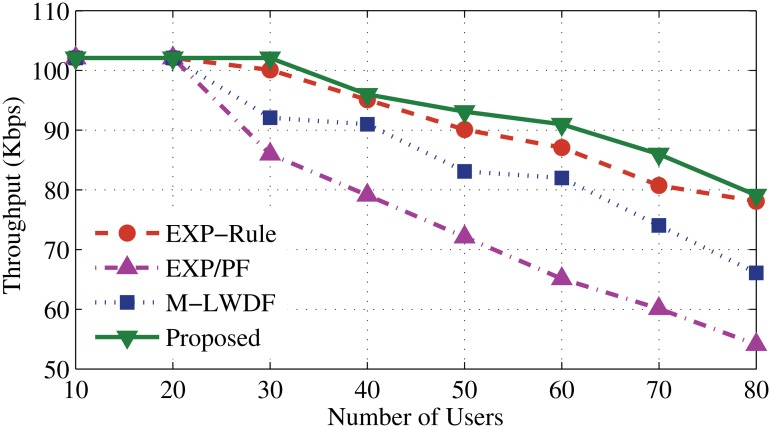
Average throughput for DA application.

**Fig 4:**
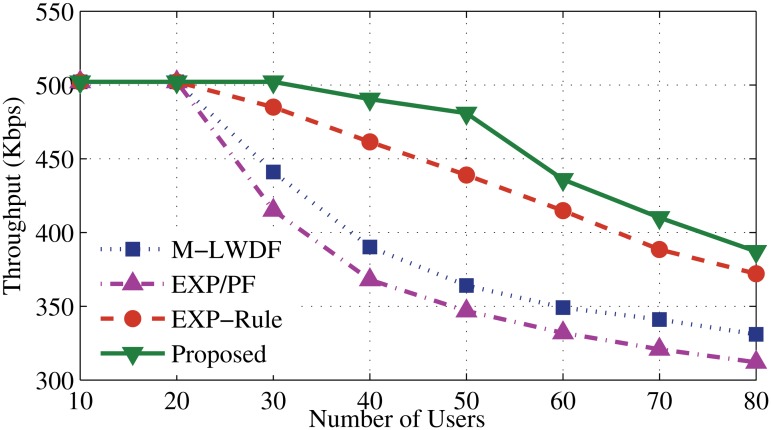
Average throughput for DER application.

Scheduling algorithms, such as EXP-Rule, M-LWDF and EXP/PF have been chosen to compare with the proposed algorithm. The new algorithm focuses on giving high priority to the RT applications and above existing algorithms are popular and well accepted. The throughput performance of the proposed algorithm is illustrated in Figs. 3 and 4. It shows a high ability to serve up to 30 users and then start degrading slowly comparing with other algorithms. Two reasons behind the robustness of the proposed algorithm are the bandwidth estimation and allocation and dynamic scheduling approach. For the bandwidth estimation and allocation, when the number of users increases (after 20 users), some resources shift from EV application as a process to keep serving the users of DA and DER. Another issue is the way of making the scheduling decision for DA and DER guarantees to provide the service based on urgency to serve. At the same time, it needs to satisfy the application’s demands (high weight coefficients for delay and instantaneous data rate) that maintains good QoS for DA and DER users along with increasing the user number.

Meanwhile, the EXP-Rule algorithm shows better performance than the M-LWDF and EXP/PF. The EXP-Rule takes the delay metric of the considered user ratio to the sum of the experienced delays of all real time users. That means it exponentially prioritizes the user which has the highest waiting head of line delay along with the higher channel condition of this user. As a result, it offers more resources to the DA and DER users than EV users. After 20 users, M-LWDF and EXP/PF sharply degrade even though M-LWDF considers the delay boundaries and channel status of the DA and DER applications. For that reason, it fails to prioritize their users when packet delay is approaching (clearly after 20 users). From Figs. 3 and 4, it can be said that the proposed algorithm shows noticeable enhancement by 1%, 10% and 15% compared to EXP-Rule, M-LWDF and EXP/PF, respectively.

**Fig 5:**
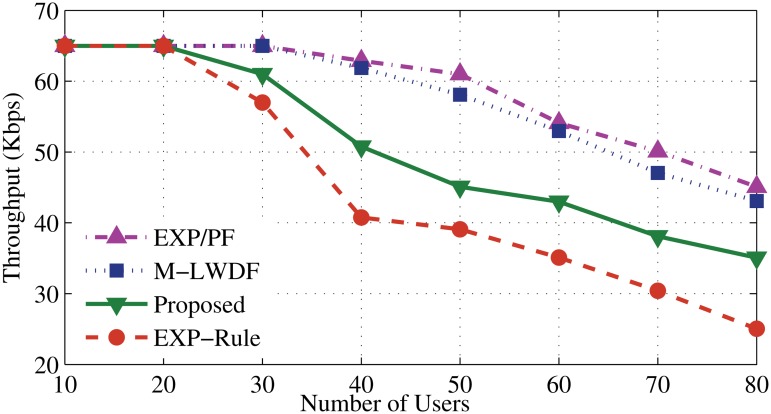
Average throughput for EV application.

For the EV application (Fig. 5), the proposed algorithm shows slightly better performance than EXP-Rule algorithm. The throughput is stable like other algorithms up to 20 users, but starts decreasing when the amount of the user increases. EXP-Rule shows the lowest performance since the delay boundary of the EV application is 2000 ms. The EXP/PF and M-LWDF show better performance than other algorithms since both of them have the PF metric, which has a direct effect on the scheduling decision. And, even though they are organized to serve the real time applications, they still need to provide the degree of service up to a certain number of users for the EV application.

**Fig 6:**
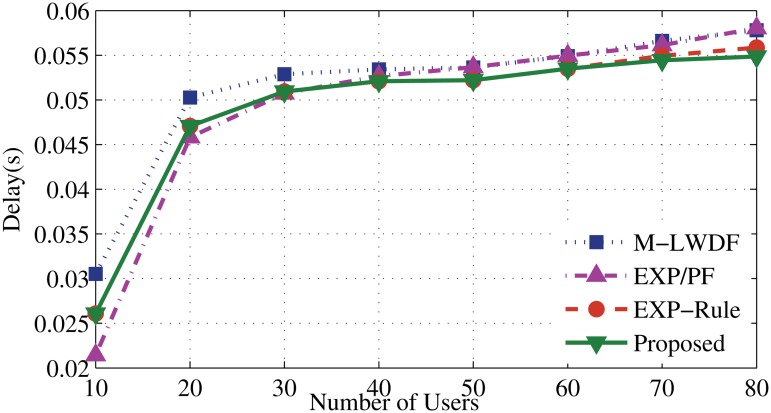
Packet delay of DA flows.

**Fig 7:**
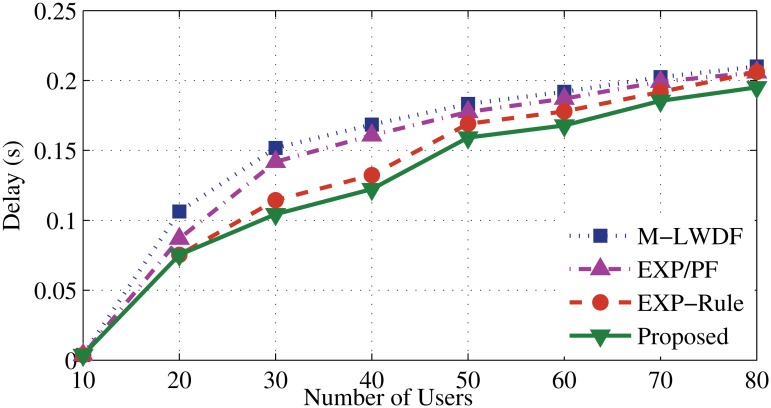
Packet delay of DER flows.

**Fig 8:**
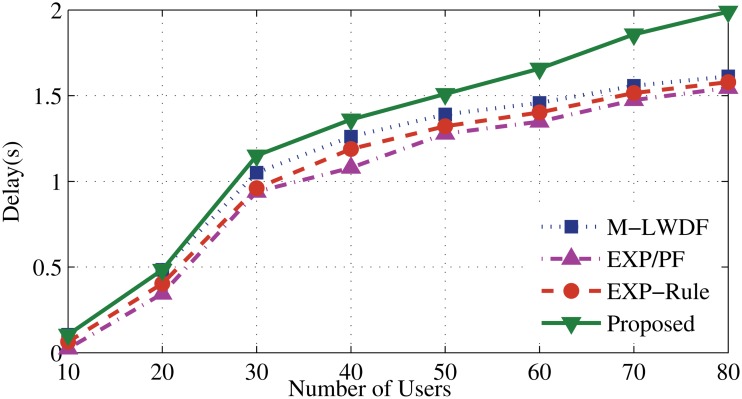
Packet delay of EV flows.

Figs. 6 and 7 illustrate that the new algorithm has the lowest serving delay compared to other scheduling algorithms. It gives higher concern to the delay metric than the other metrics. Once the number of RT users increases (after 20 users), the EV application experiences degradation of the servicing level. That leads to add more delay until the higher priority users have been served (as described in Fig. 8). The EXP-Rule shows an average level of delay since it concerns about maintaining the delay of DA and DER users within the acceptable range even if EV’s users do not receive enough services. The M-LWDF and EXP/PF show the higher delay level for DA and DER users as the PF metric at both approaches forces to provide the services for the N-RT user. As a result, more waiting time for the RT packets will be added as some resources are shifted to serve the EV users.

**Fig 9:**
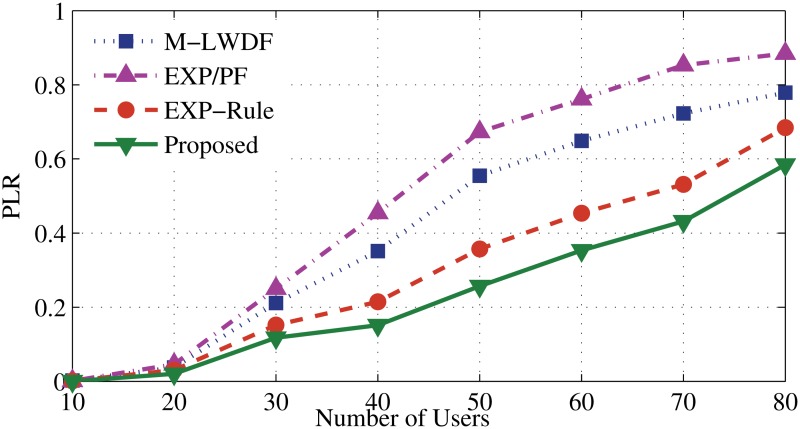
Packet loss rate of DA flows.

In Fig. 9, the proposed algorithm shows the lowest packet loss rate (PLR), which proves that new algorithm has the ability to maintain good quality of service for DA user. And, this ratio shows relatively low growth for the proposed algorithm compared to other algorithms. At 80th user, the PLR of the proposed algorithm shows improved performance up to 5%, 10% and 15% compared to EXP-Rule, M-LEDF and EXP/PF, respectively.

**Fig 10:**
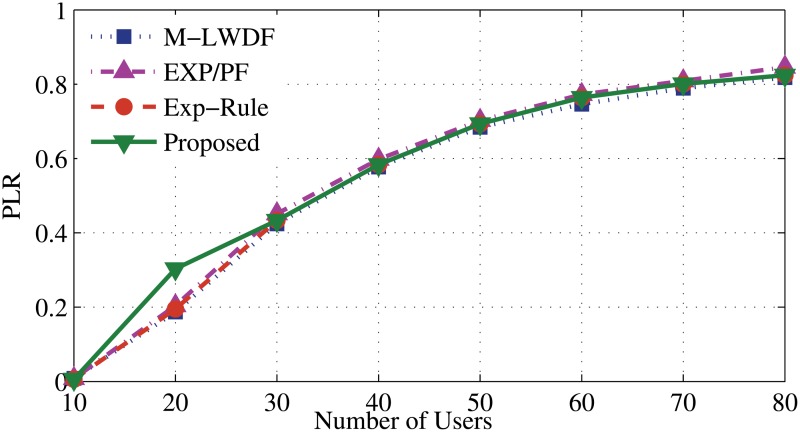
Packet loss rate of DER flows.

**Fig 11:**
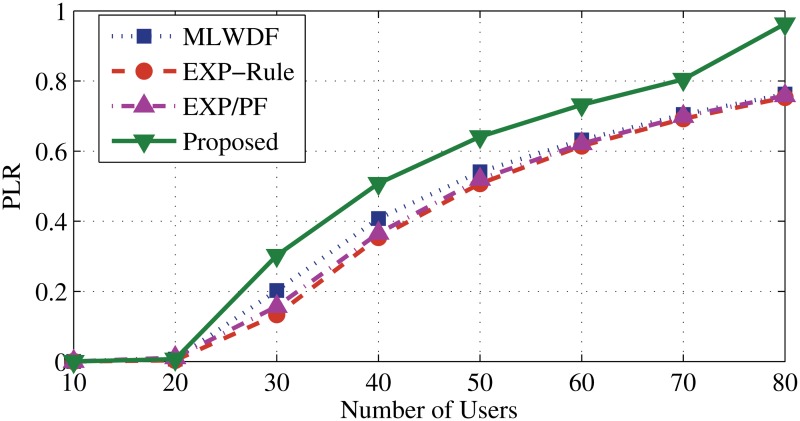
Packet loss rate of EV flows.

For DER flows in Fig. 10, all scheduling algorithms show almost same level of PLR. Fig. 11 shows that the proposed algorithm has the highest PLR due to positive probability. EV application drops the packets as a scarifying process to serve the real time user.

**Fig 12:**
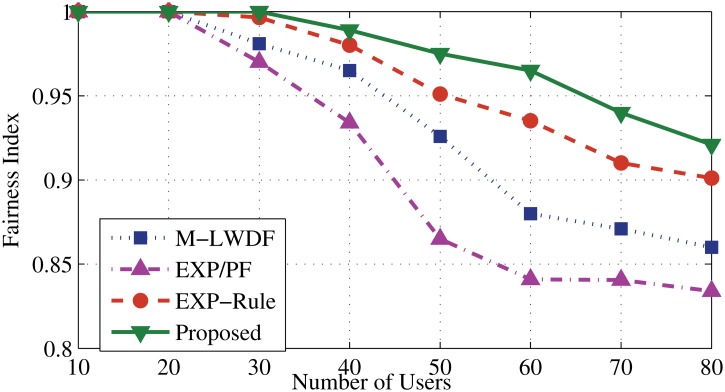
Average Packet Fairness Index for all users.

Average Packet Fairness Index among all users is illustrated in Fig. 12. As the scheduling metric is following the same method for serving the users, the proposed algorithm shows higher performance compared to other algorithms. It reaches to 0.93 even in overloaded situations (up to 80 users), followed by EXP-Rule which is 0.9. Whereas, the M-LWDF and EXP/PF show less contrast by 0.86 and 0.84 fairness index, respectively at the same number of users.

## Limitations and Future Work

The main objective of the proposed algorithm is to distribute the RBs in an efficient way based on the urgency of the specific applications. In some cases, scheduler takes all or most of the RBs from lower priority application to serve the higher priority application. This causes a longer wait or denial of services for the lower priority application.

As a future work, the scheduling approach can be extended by introducing more criteria, such as queue length in terms of the scheduling decision. It can also be adjusted to ensure the minimum level of service for lower priority application. Our next goal is to modify the proposed method to make it useful to serve a variety of applications with different goals, such as elastic resources and shared services scenario. It will be based on migrating the cloud computing system with SG applications, which will guarantee high robustness in terms of resistance and privacy protection and hardware failure issues. We will test it in different networks, such as LTE-Advanced.

## Conclusion

This paper has focused on three smart grid applications, namely DA, DER and EV. These applications can be useful for supporting the grid with renewable energy, such as wind and solar over LTE networks. The novel bandwidth estimation and allocation technique and the new scheduling algorithm for each class are proposed as a guarantee for the efficient utilization of available network resources that satisfied the application’s demand. The bandwidth estimation is based on giving the satisfied amount of resources for each application if there is sufficient bandwidth; otherwise, the resources will be distributed according to the priority of each class. Whereas, the scheduling algorithm uses dynamic scheduling technique for three criteria, namely delay, past average throughput and instantaneous transmission rate. It demonstrates its ability to provide a robust solution for solving the users’ scheduling issues with respect to SG application QoS’s demands. Simulation results prove that, the proposed technique achieves higher throughput, lower delay and lower packet loss rate for DA, DER applications as well as provides a degree of service for EV application. The proposed algorithm shows noticeable improvement for DA and DER applications by 1%, 10% and 15% compared to EXP-Rule, M-LWDF and EXP/PF, respectively.
